# Validation of Difficult Airway Physiological Score (DAPS) in Critically Ill Adults Undergoing Endotracheal Intubation in the Emergency Department

**DOI:** 10.1155/2024/6600829

**Published:** 2024-04-23

**Authors:** Shahan Waheed, Junaid Abdul Razzak, Nadeem Ullah Khan, Ahmed Raheem, Asad Iqbal Mian

**Affiliations:** ^1^Department of Emergency Medicine, Aga Khan University, Karachi, Pakistan; ^2^Department of Emergency Medicine, New York Presbyterian Weill Cornell Medicine, New York, USA

## Abstract

**Background:**

Critically ill patients have increased risk of cardiovascular collapse following endotracheal intubation due to physiological instability. This study aims to validate the Difficult Airway Physiological Score (DAPS) in adults to predict the risk of serious outcomes in the emergency department of a tertiary care private hospital.

**Methods:**

This is a cohort study conducted in the emergency department (ED) from 2021 to 2022. Difficult Airway Physiological Score (DAPS) was derived from a sample of 1021 patients through a retrospective study. The variables in the score were age, gender, time of intubation, vitals and vomiting at presentation, pH <7.3, fever, physician's anticipation for patient decline, and agitation. The model performance was assessed prospectively on a separate dataset (*n* = 326) using train-test split method. Postintubation desaturation, hypotension, cardiac arrest, and mortality postintubation were the serious outcomes. ROC analysis, sensitivity, specificity, PPV, and NPV were used to assess score validity.

**Results:**

Our study includes 326 patients, of which 123 (37.7%) were males and 203 (62.2%) were females. The sample was divided into high-risk (DAPS ≥10) group, *n* = 194 with mean age of 52 (SD = ±18) years, and low-risk (DAPS <10) group, *n* = 132 with mean age of 47.7 (SD = ±17.4) years. The shock index ≥0.9 was in 128 (66%), while it was <0.9 in low-risk *n* = 111 (84%), *p* value <0.001. Similarly, pH <7.3 was seen in 70 (36.1%) in high-risk group compared to 4 (3%) in low-risk group, *p* value <0.001. Cardiac arrest was observed in 56 (17.2%) patients, of which 45 (23.2%) were in high-risk and 11 (8.3%) in low-risk groups (*p* < 0.001). Hypotension was the primary outcome in the high-risk group 100 (51.5%) versus 32 (24.2%) in low-risk group (*p* < 0.001). The DAPS of 10 had an area under the curve of 0.865 (0.71–0.84). The sensitivity of DAPS was 78.5%, specificity 77.9%, and accuracy 78.2%.

**Conclusion:**

The score can accurately predict serious outcomes in critically ill adult patients with physiologically difficult airway demonstrating good sensitivity and specificity.

## 1. Background

The proportion of difficult intubation ranges from 10% to 27% in emergency department compared to 1% to 9% in the operating room [1]. Studies show 28% of acutely ill patients undergoing endotracheal intubation experience life-threatening complications like hypoxemia, hypotension, airway trauma, and cardiac arrest [2, 3]. Furthermore, the incidence of difficult intubation is significantly increased in the emergency department due to critical illness, which increases the risk of adverse events [4]. Endotracheal intubation in a critically ill adult with physiological instability is associated with many anticipated and unanticipated challenges [5]. The physiologically difficult airway is defined as an airway in which an adult with severe physiological abnormalities (hypotension—defined as systolic blood pressure <90 mmHg, severe metabolic acidosis—defined as pH <7.3, and hypoxia—defined as peripheral oxygen saturation <92% and right heart failure) had an increased risk of cardiovascular collapse or mortality after intubation or during transition to positive pressure ventilation [5, 6].

The data for successful intubation and peri-intubation complications are limited from developing countries [7]. Early identification of physiologically difficult intubation can help clinicians attempt early stabilization and hence reducing the risk of serious outcomes like hypotension, cardiac arrest, mortality, and hypoxia. However, unlike anatomically difficult airways, there is a paucity of scores that can predict serious outcomes in physiologically difficult airways [8–10]. Three scores are currently reported in the literature that addresses physiological instability variables in their difficult airway assessment scores [11–13]. These scores have several limitations like utilizing prehospital sample, focus on only hypotension or hypoxia as a predictor of difficult airway, and assessing exhaustive laboratory parameters, which make it use cumbersome in the emergency department [11–13].

Therefore, there is a need to derive and validate a score that should address physiological predictors in a critically ill adult patient undergoing endotracheal intubation to predict risk of serious outcomes. The goal of this investigation is to validate the physiological difficult airway score to predict serious outcomes among critically ill adults needing endotracheal intubation in the emergency department.

## 2. Methods

### 2.1. Study Design and Setting

This is a prospective cohort validation of DAPS at the emergency department of Aga Khan University Hospital from 2021 to 2022. The recruiting center is an urban, academic 62-bedded emergency department that receives 60,000 patients annually. The inclusion criteria were all adult patients (≥18 years) who presented to the ED and require endotracheal intubation. Patients with oropharyngeal tumors that require advance airway measures due to the distorted anatomy, patients with out-of-hospital cardiac arrest or ongoing CPR, and pregnant females due to the varied physiological derangements were excluded from the study. The criteria for intubation were severe respiratory distress, worsening hypoxia not responding to noninvasive positive pressure ventilation, Glasgow coma scale less than 8, anticipated decline (intubation based on physician discretion), and impending airway compromise. We estimated our sample size to be 268 based on absolute precision of 6% with 95% confidence interval and at 5% level of significance. The sample size was calculated from a study by Smischney et al. by WHO calculator showing a 52% rate of postintubation hypotension [14].

### 2.2. Data Collection

Patients requiring intubation in the ED were identified either at the triage or by the resuscitation room physicians, who subsequently informed the research assistant. The research assistant screened patients after taking a verbal consent either from the patient (with intact capacity, which was understanding, appreciation, reasoning, and expression of choice with regard to the process that was followed in the study) or the accompanying decision maker, which was later followed by a written consent. The pre-intubation vitals at the triage along with demographic variables were gathered during assessment. The research assistant did not interfere in the management of the patients needing endotracheal intubation. The data were collected on a pretested questionnaire. The pretest was done on 10 questionnaires, which were not included in the final analysis. The data collected on the form were reviewed by the physician who was involved in endotracheal intubation to review any missing data. Presentation symptoms, presentation vital signs, reason for intubation, difficult airway assessment, drugs used in endotracheal intubation, and other procedural data were collected. The questionnaires were periodically reviewed by the principal investigator for accuracy. Follow-up of the intubated patients was done in the ED at 15 minutes and 1 hour postintubation for record of vitals. The estimated risk for serious outcomes for different DAPS categories was not mentioned on the questionnaire, to prevent physicians from making disposition decisions based on the risk score. To maintain good reporting practice, the TRIPOD checklist was used [15].

### 2.3. Serious Outcomes

The primary outcomes were hypotension (defined as a drop in systolic blood pressure <90 mmHg) and hypoxia (defined as oxygen desaturation <92% within 1 hour of intubation). The secondary outcomes were cardiac arrest (defined as the absence of pulse after endotracheal intubation in the emergency department) and mortality (defined as death occurring within 1 hour after intubation in the emergency department). All the above outcomes were measured at different points in time: immediately postintubation, at 15 minutes, and at 1 hour postintubation.

### 2.4. Statistical Analysis

Data were entered into the Redcap, and data collection and data entry functions were analyzed using SPSS-22 (IBM, IL, USA) and Python 3.8.14. We describe the study patients using means, ranges, and standard deviations as appropriate for continuous variables and frequencies with proportion for categorical variables. Shapiro–Wilk's test was applied to check the quantitative variable's age normality. The *χ*^2^ test was used to compare proportions. The association among hypotension, desaturation, cardiac arrest, mortality, and various demographic, clinical, and laboratory characteristics was evaluated using the chi-square or Fisher's exact test or an independent sample *t* test or Mann–Whitney *U* test, as appropriate. The prospective validity of the Difficult Airway Physiological Score was determined by plotting the ROC curve with 95% confidence interval (CI). The major discriminating point of the DAP score was established by computing Youden's J statistic, sensitivity, specificity, positive predictive value (PPV), and negative predictive value (NPV) with 95% confidence intervals at different score thresholds. A *p* value of 0.05 was considered statistically significant in all analyses.

### 2.5. Derivation of the Score

The score was retrospectively derived through a sample of 1021 patients who had endotracheal intubation from January 2016 to December 2020. The sample was divided into development and validation datasets by the train-test split method. A total of 812 (80%) samples were randomly assigned to the development dataset and the remaining 209 (20%) to the validation dataset. Distribution of data on 38 selected system-related and patient-related factors was not statistically different between the development and validation groups, respectively. In the modeling–fitting phase, 27 significant independent predictors for physiological difficult airway were identified. The identified factors were taken with adjusted OR (95% CI). The final 12-factor model consists of female gender (1.49 [1.07–2.07], point = 1), age ≥45 years (1.58 [1.13–2.19], points = 2), time of intubation (2.12 [1.55–2.9], points = 2), presentation hypotension (1.99 [1.19–3.34], points = 2), presentation respiratory distress (1.68 [1.17–2.42], points = 2), vomiting (3.41 [2.17–5.35], points = 3), pH <7.3 (4.39 [3.17–6.09], points = 4), shock index ≥0.9 (1.68 [1.33–2.57], points = 2), fever (2.13 [1.38–3.3], points = 2), anticipated decline (1.89 [1.16–3.06], points = 2), GCS <15 (1.66 [1.14–2.41], points = 2), and agitation (1.42 [1–2.02], points = 1). The Difficult Airway Physiological Score is shown in Table 1. Additional information regarding the derivation of the score can be found in the published manuscript [16].

## 3. Results

### 3.1. Patient Characteristics

**Figure 1:**
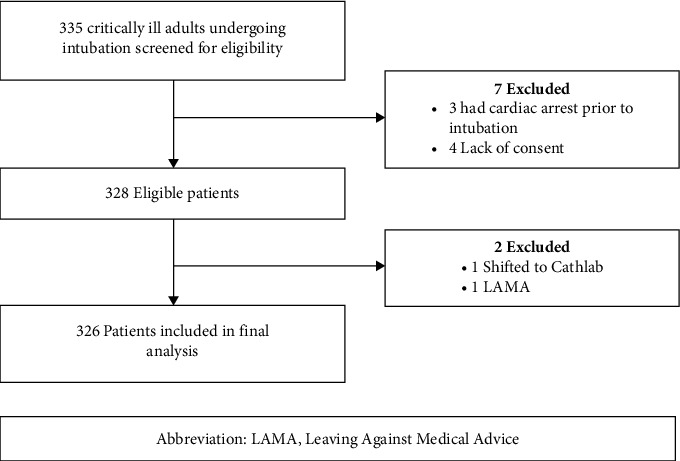
Study patients flow through screening and enrollment.

A total of 335 patients were eligible to participate in the study, of which 326 patients were included in the final analysis. The enrollment of study participants is shown in Figure 1. Nine patients were excluded due to cardiac arrest prior to intubation, lack of consent because of lack of capacity by the patient or unwilling to participate in the study by the decision maker, leaving against medical advice, and immediately shifting to other areas.

The characteristics of the study participants included in our study are shown in Table 2. Of 326 patients eligible for the study, 123 [37.7%] were males with a mean age of 50.3 (SD = ± 17.8) years. Most of them have age >45 years, 198 [60.7%]. 134 [41.1%] intubations happened at night (10 pm–08 am), with shortness of breath being the most prevalent symptom, 153 [46.9%], followed by drowsiness (GCS <15), 96 [29.4%]. The median systolic blood pressure at ED presentation was 128 mmHg (106–150) and diastolic blood pressure 76 mmHg (60–90), heart rate 110 beats per minute (90–125), and oxygen saturations 93% (82–98). Respiratory distress was the most common reason for intubation, 239 [73.3%], followed by coma, 220 [67.5%], and anticipated decline, 143 [43.9%]. Majority of patients have shock index of <0.9 (*n* = 177, 54.3%) and pH ≥7.3 (*n* = 252, 77.3%).

### 3.2. Risk Stratification

The Difficult Airway Physiological Score was used to divide our study sample into high risk (DAPS ≥10) 194, mean age of 52 (SD = ± 18) years, and low risk (DAPS <10) 132, with a mean age of 47.7 (SD = ± 17.4) years. The study sample division as per Difficult Airway Physiological Score is shown in Table 3. Majority of the patients were falling in the age group of ≥45 years in both high- and low-risk groups. The association between age group and risk group was found to be highly significant (*p* value<0.001). Many of the intubations were performed in the morning hours (8AM to 4PM), 66 [34%], followed by evening (4PM to 10PM), 64 [33%] in high-risk group, while it was 70 [53%] in low risk where most of the intubations were performed at night (10PM–8AM), *p* value <0.001. The main reason for intubation in high-risk group was respiratory distress 169 [87.1%], followed by coma, 132 [68%], and hypoxia, 101 [52.1%]. On the contrary, in the low-risk group, the indication was coma, 88 [66.7%], followed by respiratory distress, 70 [53%], and anticipated decline, 59 (44.7%). The shock index was ≥0.9, which was observed in 128 [66%], whereas it was <0.9 in majority in the low-risk group, 111 (84.1%), *p* value <0.001. In Difficult Airway Physiological Score ≥10, pH was <7.3 in 70 [36.1%] compared to 4 [3%] in the score of <10, *p* value <0.001.

### 3.3. Outcome Analysis

Hypotension was the most observed serious event among patients undergoing endotracheal intubation 132 [40.5%], followed by death within 1 hour after intubation in 82 [25.2%] and oxygen saturation <92% in 80 [24.5%]. Risk stratification of serious outcomes as per the Difficult Airway Physiological Score is shown in Table 4.

Cardiac arrest was present in 56 [17.2%], of which 45 [23.2%] was in high risk and 11 [8.3%] were in low risk, *p* value <0.001. Hypotension was the most serious outcome in the high-risk group 100 [51.5%] compared to 32 [24.2%] in low-risk group, *p* value <0.001. Desaturation was observed more in high-risk group 60 [30.9%] compared to low-risk group 20 [15.2%], *p* value 0.001. The blood pressure and oxygen saturation trends as per the Difficult Airway Physiological Score showed a major drop in both systolic and diastolic blood pressures in the high-risk group. The systolic blood pressure, diastolic blood pressure, and oxygen saturation trends between high-risk and low-risk groups as per DAPS with *p* value are shown in Appendix 2 as supplementary Figure 1.

### 3.4. Sensitivity Analysis

**Figure 2:**
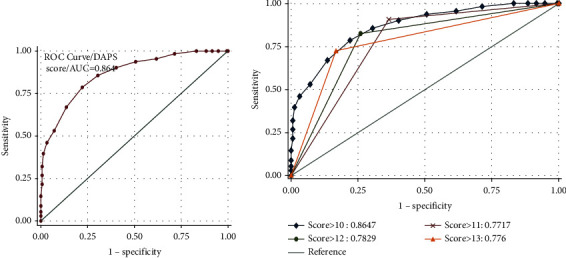
(a) Receiver operating characteristic (ROC) curve for the Difficult Airway Physiological Score (DAPS) with an area under the curve at 0.864 and (b) receiver operating characteristic (ROC) curve showing the area under the curve at different score thresholds.

In the ROC curve analysis, the AUC of the DAPS was found to be 0.864 (95% CI 0.71–0.84) as shown in Figure 2(a). In the ROC curve estimation of different score thresholds, a DAPS of >10 has a significant area under the curve compared to others as demonstrated in Figure 2(b). Good relation was observed between the observed versus the predicted rate of physiological difficult airway high risk/low risk in the development dataset as per the Hosmer–Lemeshow goodness of test by decile of predicted risk.

The optimal cutoff value for the DAPS was found to be a score of ≥10, at which sensitivity of 78.5% (71.7% to 84%), specificity of 77.9% (70.7% to 83.8%), PPV of 80% (81.20% to 91.19%), NPV of 76% (50.37% to 65.53%), and accuracy of 78.2% were observed. The sensitivity analysis table of the score with the score threshold value at different score cutoffs is shown in Appendix 1 as supplementary Table 1.

The total number of true positives and true negatives at a score >10 was 78% as shown in the heat map in Appendix 3 as supplementary Figure 2.

## 4. Discussion

In this single-center cohort study, we validated the Difficult Airway Physiological Score on a new cohort of patients who underwent endotracheal intubation in the emergency department. Our study confirmed the accurate model performance characteristics of the original decision tool. The 12-variable score is easy to use and relevant to the current practice of emergency medicine with special consideration to low-middle-income countries where the burden of critically ill patients is massive due to a weak primary healthcare system. The results of our study showed that patients with score of 10 or more are at high risk of having serious outcomes postintubation in the emergency department. In our validation study, the Difficult Airway Physiological Score showed a satisfactory discriminative power with high sensitivity and specificity. The AUC for the DAPS was 0.864, which shows a need to pay particular attention to these patients requiring intubation in the emergency department. The study results emphasize the importance of a priori resuscitation in such cases as it will make the situation worse on intubation. However, there will be instances where crash intubation is done in the resuscitation room and endotracheal intubation is performed at the earliest.

Postintubation hypotension is a well-known complication of endotracheal intubation that is reported in the literature [4, 13, 14]. It is associated with high mortality and extended ICU care. Therefore, timely assessment and intervention is paramount [17]. Our score demonstrates that with an increasing score, the risk of serious outcomes increases. The sensitivity analysis reveals a cutoff score of 10 based on which we have divided the cohort into high risk and low risk. This has resulted in a significantly high proportion of patients exhibiting physiologically difficult airway having serious outcomes on endotracheal intubation. The reason can be due to increase in presentation of critically ill patients, poor primary healthcare system, and healthcare cost borne by the patient, which results in delayed presentation. Furthermore, being a private healthcare setup, the presentation of the patients is late, which results in increase severity of disease on emergency department presentation. Postintubation hemodynamic instability is a commonly reported occurrence in the ICU setting as well, in which disease severity parameters tend to dominate [5]. The incidence of postintubation hypotension in ICUs is reported between 20% and 46% for critically ill patients, who are associated with poor outcomes when intubated [18]. Additionally, our study findings are in accordance with hypotension prediction tool, which states that peri-intubation hypotension increases the risk for adverse clinical events [13]. This study was conducted in the ICU, and the tool validity in emergency setting was not done. Advanced age is one of the predictors, and majority of the cohort with a score of 10 and above had age of 45 years and above. Moreover, postintubation cardiac arrest is common in our cohort of patients. One possible explanation is that these individuals were recruited during the COVID pandemic, when the percentage of critically ill COVID patients was high and serious outcomes were more prevalent. Due to a lack of ICU beds, these patients had to wait for several hours in the emergency department after being intubated; as a result, the emergency room seems to have high frequency of serious outcomes, which may be connected to their illness process.

The unanticipated difficult endotracheal intubation is a common occurrence in the emergency room and a major source of concern for both the emergency attendings and anesthesiologists [3, 19, 20]. It is hence incumbent to identify a score that is quick, easy to apply, and equally sensitive and specific, to accurately predict serious outcomes in potentially physiologically difficult endotracheal intubations. The physiological disturbances that are evaluated in our sample were hypotension, hypoxemia, shock index, and pH. The evaluation of right heart failure, which is one of the variables associated with decompensation, was not conducted due to the variability in bedside ultrasonography that relies on the operator's skills. Additionally, the use of point-of-care ultrasound (POCUS) in our environment is still in the early stages of development. The strengths of this study include the systematic data collection and prior plan to minimize anticipated bias due to its prospective nature. Additionally, this is the first study that focuses physiological instability variable assessment in the emergency department. Our score is practical in the daily clinical practice as it is based on presentation clinical variables and a single point-of-care test, which should not have a negative effect on the time management in such difficult scenarios.

In summary, this is one of the few studies that have investigated the physiological variables and their association in predicting serious outcomes in critically ill adult patients in the emergency department. The data are from a tertiary care hospital of a developing world emergency department that is a valuable addition to know the patient presentations in a developing world emergency department. We believe that a patient with physiological instability identified early and subsequently managed may result in improved patient outcomes and better patient safety. A multicenter validation study is needed to evaluate its use as an adjunct in predicting serious outcomes among patients with physiological instability in the emergency department.

### 4.1. Limitations

There were several limitations in our study. First, it is a single-center study and will need external validation through a multicenter study. Second, the data collection of the study coincided with the COVID-19 pandemic, and there were some changes in the intubation norm like the use of video laryngoscopy, use of respirators and face shield, increased prevalence of resistant hypoxia, and changes in bag-mask ventilation techniques that have significantly influenced the intubation process but could not be determined precisely and were not factored in this study. Third, the study did not collect data on direct long-term consequences of adverse peri-intubation events on specific patient's outcomes (e.g., hypoxic brain injury). However, the aim of the study was to prospectively collect data on immediate adverse events. Fourth, interpretation of results may be biased by residual or unmeasured confounders (drugs for rapid sequence intubation, comorbid, and disease severity status). The residual confounders may have influenced the higher incidence and severity of adverse events in some subgroups of critically ill patients. We tried to control these confounders through our statistical analysis. Lastly, our study did not investigate DAPS and its impact on clinical care to improve patient's outcome.

## 5. Conclusion

The Difficult Airway Physiological Score (DAPS) was validated to predict the risk of serious outcomes among critically ill adult patients undergoing endotracheal intubation in the emergency department. Our score demonstrates good sensitivity, specificity, and accuracy in predicting the risk of serious outcomes on patients with physiological difficult airway.

## Figures and Tables

**Table 1:** The difficult airway physiological score.

Category	Points	Beta coefficient (*β*)
Female gender	1	0.38
Age ≥45 years	2	0.44
Time of intubation/shift duty (morning/evening)	2	0.77
Presentation hypotension (<90 mmHg)	2	0.68
Presentation respiratory distress	2	0.50
Vomiting	3	1.22
Shock index ≥0.9	2	0.86
pH <7.3	4	1.86
Fever	2	0.78
Anticipated decline	2	0.51
GCS <15	2	0.59
Agitation	1	0.36

**Table 2:** Characteristics of patients having endotracheal intubation in the emergency department (*n* = 326).

Demographic and clinical factors	*n* (%)
*Age*	
<45 years	128 [39.3%]
≥45 years	198 [60.7%]

*Gender*	
Male	123 [37.7%]
Female	203 [62.2%]

*Triage vitals on presentation * ^*∗*^	
Systolic blood pressure	128 (106–150)
Diastolic blood pressure	76 (60–90)
Heart rate	110 (90–125)
Oxygen saturations	93 (82–98)
Respiratory rate	28 (22–36)

*Presenting complaint*	
Shortness of breath	153 [46.9%]
Fever	94 [28.8%]
Drowsiness	96 [29.4%]
Seizures	20 [6.1%]
Trauma	33 [10.1%]
Coma	51 [15.6%]
Others	135 [41.4%]

*Reasons for intubation*	
Coma	220 [67.5%]
Hypoxia	130 [39.9%]
Metabolic acidosis	89 [27.3%]
Anticipated decline	143 [43.9%]
Respiratory distress	239 [73.3%]
Polytrauma	17 [5.2%]
Isolated trauma	21 [6.4%]
Gunshot injury	4 [1.2%]
Others	8 [2.5%]

*Shock index*	
<0.9	177 [54.3%]
≥0.9	149 [45.7%]

*pH group*	
>7.3	252 [77.3%]
≤7.3	74 [22.7%]

^*∗*^Median (Interquartile Range).

**Table 3:** Emergency department intubation characteristics among patients as per difficult airway physiological score status.

Characteristics	Risk stratification as per DAPS		*p* value
	High risk ≥10 (194)	Low risk <10 (132)	
Age (years)	52 (SD = ± 18)	47.7 (SD = ± 17.4)	<0.001^*∗*^

*Age groups*			
<45 years	68 [35.1%]	60 [45.5%]	<0.059
≥45 years	126 [64.9%]	72 [54.5%]	

*Gender*			
Male	126 [64.9%]	77 [58.3%]	0.226
Female	68 [35.1%]	55 [41.7%]	

*Shift*			
Morning (8AM–4PM)	66 [34%]	37 [28%]	<0.001^*∗*^
Evening (4PM–10PM)	64 [33%]	25 [18.9%]	
Night (10PM–8AM)	64 [33%]	70 [53%]	

*Triage presentation vitals (median IQR)*			
Systolic blood pressure (mmHg)	120.5 (140–94)	142 (167–119)	<0.001^*∗*^
Diastolic blood pressure (mmHg)	70 (87–53)	81 (94–70)	<0.001^*∗*^
Heart rate	118 (130–102)	96 (112–78)	<0.001^*∗*^
Oxygen saturations (%)	90.5 (97–73)	98 (99–89)	<0.001^*∗*^
Respiratory rate	30 (36–24)	24 (31.5–20)	<0.001^*∗*^

*Reason/s for intubation*			
Coma	132 [68%]	88 [66.7%]	0.795
Hypoxia	101 [52.1%]	29 [22%]	0.046^*∗*^
Metabolic acidosis	71 [36.6%]	18 [13.6%]	<0.001^*∗*^
Anticipated decline	84 [43.3%]	59 [44.7%]	0.803
Respiratory distress	169 [87.1%]	70 [53%]	<0.001^*∗*^
Polytrauma	7 [3.6%]	10 [7.6%]	0.114
Isolated trauma	5 [2.6%]	16 [12.1%]	0.025^*∗*^
Gunshot injury	2 [1%]	2 [1.5%]	0.017^*∗*^
Others	5 [2.6%]	3 [2.3%]	0.861

*Shock index*			
<0.9	66 [34%]	111 [84.1%]	<0.001^*∗*^
≥0.9	128 [66%]	21 [15.9%]	

*pH*			
≥7.3	124 [63.9%]	128 [97%]	<0.001^*∗*^
<7.3	70 [36.1%]	4 [3%]	

*Drugs used for intubation*			
Ketamine	59 [30.4%]	14 [10.6%]	<0.001^*∗*^
Propofol	61 [31.4%]	61 [46.2%]	0.007^*∗*^
Etomidate	3 [1.5%]	1 [0.8%]	0.525
Midazolam	87 [44.8%]	66 [50%]	0.360
Succinylcholine	87 [44.8%]	81 [61.4%]	0.003^*∗*^
Rocuronium	23 [11.9%]	7 [5.3%]	0.045^*∗*^

*Heaven criteria*			
Hypoxemia	94 [48.5%]	34 [25.8%]	<0.001^*∗*^
Extremes of size	19 [9.8%]	8 [6.1%]	0.230
Anatomic abnormalities	30 [15.5%]	16 [12.1%]	0.395
Vomit/blood/fluid	58 [29.9%]	53 [40.2%]	0.055
Exsanguination	28 [13.2%]	17 [14.9%]	0.670
Neck mobility issues	25 [12.9%]	20 [15.2%]	0.561

^*∗*^donates statistical significance at the level (*p* < 0.05).

**Table 4:** Risk stratification of serious outcomes as per difficult airway physiological score.

Outcome	All (*n* = 326)	Risk stratification as per DAPS		*p* value
		High-risk score ≥10 (*n* = 194)	Low-risk score <10 (*n* = 132)	
Cardiac arrest	56 [17.2%]	45 [23.2%]	11 [8.3%]	<0.001^*∗*^
Hypotension (SBP <100 mmHg)	132 [40.5%]	100 [51.5%]	32 [24.2%]	<0.001^*∗*^
Desaturation (<92%)	80 [24.5%]	60 [30.9%]	20 [15.2%]	0.001^*∗*^
Death in emergency (within 1 hour after intubation)	82 [25.2%]	61 [31.4%]	21 [15.9%]	0.002^*∗*^
Death in hospital	68 [20.9%]	43 [22.2%]	25 [18.9%]	0.482
Discharge	114 [35%]	55 [28.4%]	59 [44.7%]	0.002^*∗*^

^*∗*^donates statistical significance at the level (*p* < 0.05).

## Data Availability

The datasets used and analyzed during the current study are available from the corresponding author upon reasonable request.

## Additional Points

*Patient and Public Involvement*. Patients and/or the public are not involved in the design, conduct, or reporting or dissemination plans of this research.

## Supplementary Materials

Supplementary Materials Supplementary Table 1: sensitivity analysis table of Difficult Airway Physiological Score (DAPS)/threshold value at different scores cutoff by Youden's Index. Supplementary Figure 1: blood pressure and oxygen saturation trends as per the difficult airway prediction score showing a major drop in both systolic and diastolic blood pressures in the high-risk group. Supplementary Figure 2: total number of true positives and true negatives is 78%.
