# Supplementary material for: Validation of Difficult Airway Physiological Score (DAPS) in Critically Ill Adults Undergoing Endotracheal Intubation in the Emergency Department
Source: Emerg Med Int. 2024 Apr 23;2024:6600829. doi: 10.1155/2024/6600829 (PMC11401705; doi:10.1155/2024/6600829)
Supplement: Supplementary Materials — Supplementary Table 1: sensitivity analysis table of Difficult Airway Physiological Score (DAPS)/threshold value at different scores cutoff by Youden's Index. Supplementary Figure 1: blood pressure and oxygen saturation trends as per the difficult airway prediction score showing a major drop in both systolic and diastolic blood pressures in the high-risk group. Supplementary Figure 2: total number of true positives and true negatives is 78%. [file 6600829.f1.zip › Supplementary Table 1.docx]

**Supplementary Table 1. Sensitivity analysis table of Difficult Airway Physiological Score (DAPS)/Threshold value at different scores cutoff by Youden’s Index.**

| **DAPS / Threshold value** | **Sensitivity** | **Specificity** | **PPV** | **NPV** | **LR+** | **TP** | **TN** | **FP** | **FN** | **Sensitivity + Specificity** | **Accuracy** |
| --- | --- | --- | --- | --- | --- | --- | --- | --- | --- | --- | --- |
| 2 | 100% [95% C.I ; 97.3 -100] | 0.6% [95% C.I ; 0 -4] | 53% | 100% | 1.01 | 172 | 1 | 153 | 0 | 1.01 | 53.1% |
| 3 | 100% [95% C.I ; 97.3 -100] | 5.2% [95% C.I ; 2.5 -10.1] | 54% | 100% | 1.05 | 172 | 8 | 146 | 0 | 1.05 | 55.2% |
| 4 | 100% [95% C.I ; 97.3 -100] | 8.4% [95% C.I ; 4.9 -14.1] | 55% | 100% | 1.09 | 172 | 13 | 141 | 0 | 1.08 | 56.7% |
| 5 | 100% [95% C.I ; 97.3 -100] | 11.7% [95% C.I ; 7.5 -17.8] | 56% | 100% | 1.13 | 172 | 18 | 136 | 0 | 1.12 | 58.3% |
| 6 | 100% [95% C.I ; 97.3 -100] | 16.9% [95% C.I ; 11.8 -23.7] | 57% | 100% | 1.20 | 172 | 26 | 128 | 0 | 1.17 | 60.7% |
| 7 | 98.3% [95% C.I ; 94.7 -99.6] | 28.6% [95% C.I ; 22 -36.2] | 61% | 94% | 1.38 | 169 | 44 | 110 | 3 | 1.27 | 65.3% |
| 8 | 95.3% [95% C.I ; 90.9 -97.7] | 38.3% [95% C.I ; 31 -46.2] | 63% | 88% | 1.55 | 164 | 59 | 95 | 8 | 1.34 | 68.4% |
| 9 | 93.6% [95% C.I ; 88.7 -96.5] | 49.4% [95% C.I ; 41.6 -57.2] | 67% | 87% | 1.85 | 161 | 76 | 78 | 11 | 1.43 | 72.7% |
| **10** | **78.5% [95% C.I ; 71.7 -84]** | **77.9% [95% C.I ; 70.7 -83.8]** | **80%** | **76%** | **3.56** | **135** | **120** | **34** | **37** | **1.56** | **78.2%** |
| 11 | 90.1% [95% C.I ; 84.6 -93.8] | 59.7% [95% C.I ; 51.8 -67.1] | 71% | 84% | 2.24 | 155 | 92 | 62 | 17 | 1.50 | 75.8% |
| 12 | 85.5% [95% C.I ; 79.3 -90] | 69.5% [95% C.I ; 61.8 -76.2] | 76% | 81% | 2.80 | 147 | 107 | 47 | 25 | 1.55 | 77.9% |
| 13 | 66.9% [95% C.I ; 59.5 -73.5] | 86.4% [95% C.I ; 79.9 -90.9] | 85% | 70% | 4.90 | 115 | 133 | 21 | 57 | 1.53 | 76.1% |
| 14 | 52.9% [95% C.I ; 45.5 -60.2] | 92.9% [95% C.I ; 87.5 -96.1] | 89% | 64% | 7.41 | 91 | 143 | 11 | 81 | 1.46 | 71.8% |
| 15 | 45.9% [95% C.I ; 38.7 -53.4] | 96.8% [95% C.I ; 92.4 -98.8] | 94% | 62% | 14.1 | 79 | 149 | 5 | 93 | 1.43 | 69.9% |
| 16 | 39.5% [95% C.I ; 32.5 -47] | 98.7% [95% C.I ; 95 -99.9] | 97% | 59% | 30.4 | 68 | 152 | 2 | 104 | 1.38 | 67.5% |
| 17 | 32% [95% C.I ; 25.5 -39.3] | 99.4% [95% C.I ; 96 -100] | 98% | 57% | 49.2 | 55 | 153 | 1 | 117 | 1.31 | 63.8% |
| 18 | 26.7% [95% C.I ; 20.7 -33.9] | 99.4% [95% C.I ; 96 -100] | 98% | 55% | 41.2 | 46 | 153 | 1 | 126 | 1.26 | 61.0% |
| 19 | 21.5% [95% C.I ; 16 -28.3] | 99.4% [95% C.I ; 96 -100] | 97% | 53% | 33.1 | 37 | 153 | 1 | 135 | 1.21 | 58.3% |
| 20 | 14.5% [95% C.I ; 10 -20.7] | 100% [95% C.I ; 97 -100] | 100% | 51% | +Inf | 25 | 154 | 0 | 147 | 1.15 | 54.9% |
| 21 | 8.7% [95% C.I ; 5.3 -14] | 100% [95% C.I ; 97 -100] | 100% | 50% | +Inf | 15 | 154 | 0 | 157 | 1.09 | 51.8% |
| 22 | 5.2% [95% C.I ; 2.7 -9.8] | 100% [95% C.I ; 97 -100] | 100% | 49% | +Inf | 9 | 154 | 0 | 163 | 1.05 | 50.0% |
| 23 | 2.9% [95% C.I ; 1.1 -6.9] | 100% [95% C.I ; 97 -100] | 100% | 48% | +Inf | 5 | 154 | 0 | 167 | 1.03 | 48.8% |
| 24 | 0% [95% C.I ; 0 -2.7] | 100% [95% C.I ; 97 -100] |  | 47% |  | 0 | 154 | 0 | 172 | 1.00 | 47.2% |

*Abbreviations: PPV Positive Predictive Value, NPV Negative Predictive Value, LR Likelihood Ratio, TP True Positive, TN True Negatives, FP False Positive, FN False Negative. Test is positive if DAPS > Threshold value*
