# Supplementary figures and images for: Validation of Difficult Airway Physiological Score (DAPS) in Critically Ill Adults Undergoing Endotracheal Intubation in the Emergency Department
Source: Emerg Med Int. 2024 Apr 23;2024:6600829. doi: 10.1155/2024/6600829 (PMC11401705; doi:10.1155/2024/6600829)

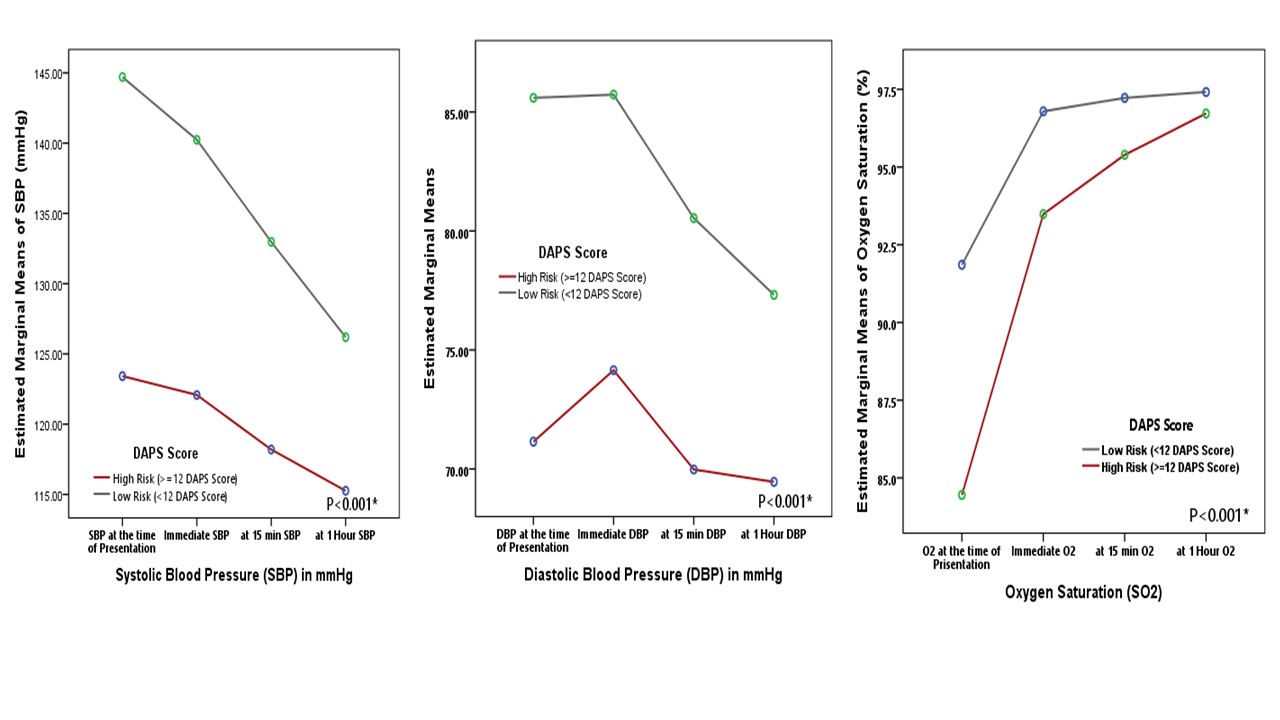

Supplement: Supplementary Materials — Supplementary Table 1: sensitivity analysis table of Difficult Airway Physiological Score (DAPS)/threshold value at different scores cutoff by Youden's Index. Supplementary Figure 1: blood pressure and oxygen saturation trends as per the difficult airway prediction score showing a major drop in both systolic and diastolic blood pressures in the high-risk group. Supplementary Figure 2: total number of true positives and true negatives is 78%. [file 6600829.f1.zip › Supplementary Figure 1.jpg]

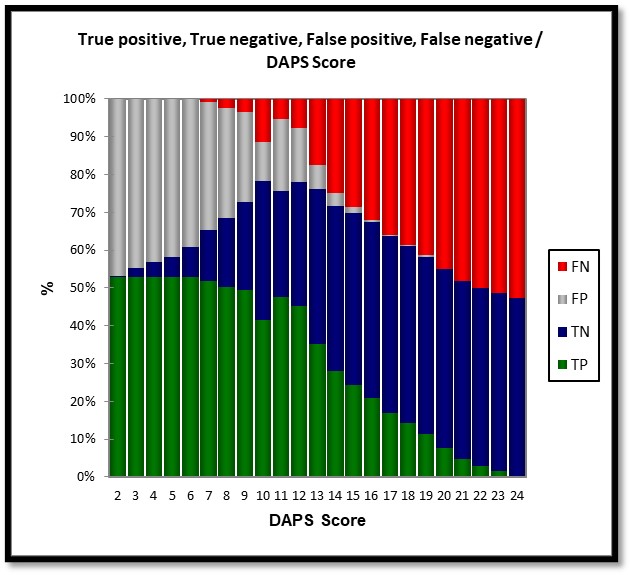

Supplement: Supplementary Materials — Supplementary Table 1: sensitivity analysis table of Difficult Airway Physiological Score (DAPS)/threshold value at different scores cutoff by Youden's Index. Supplementary Figure 1: blood pressure and oxygen saturation trends as per the difficult airway prediction score showing a major drop in both systolic and diastolic blood pressures in the high-risk group. Supplementary Figure 2: total number of true positives and true negatives is 78%. [file 6600829.f1.zip › Supplementary Figure 2.jpg]
